# Differences in Muscle and Adipose Tissue Gene Expression and Cardio-Metabolic Risk Factors in the Members of Physical Activity Discordant Twin Pairs

**DOI:** 10.1371/journal.pone.0012609

**Published:** 2010-09-16

**Authors:** Tuija Leskinen, Rita Rinnankoski-Tuikka, Mirva Rintala, Tuulikki Seppänen-Laakso, Eija Pöllänen, Markku Alen, Sarianna Sipilä, Jaakko Kaprio, Vuokko Kovanen, Paavo Rahkila, Matej Orešič, Heikki Kainulainen, Urho M. Kujala

**Affiliations:** 1 Department of Health Sciences, University of Jyväskylä, Jyväskylä, Finland; 2 Department of Biology of Physical Activity, University of Jyväskylä, Jyväskylä, Finland; 3 VTT Technical Research Centre of Finland, Espoo, Finland; 4 Department of Medical Rehabilitation, Oulu University Hospital, Oulu, Finland; 5 Department of Public Health and Institute for Molecular Medicine, University of Helsinki, Helsinki, Finland; 6 National Institute for Health and Welfare, Helsinki, Finland; Institute of Preventive Medicine, Denmark

## Abstract

High physical activity/aerobic fitness predicts low morbidity and mortality. Our aim was to identify the most up-regulated gene sets related to long-term physical activity vs. inactivity in skeletal muscle and adipose tissues and to obtain further information about their link with cardio-metabolic risk factors. We studied ten same-sex twin pairs (age range 50–74 years) who had been discordant for leisure-time physical activity for 30 years. The examinations included biopsies from *m. vastus lateralis* and abdominal subcutaneous adipose tissue. RNA was analyzed with the genome-wide Illumina Human WG-6 v3.0 Expression BeadChip. For pathway analysis we used Gene Set Enrichment Analysis utilizing active vs. inactive co-twin gene expression ratios. Our findings showed that among the physically active members of twin pairs, as compared to their inactive co-twins, gene expression in the muscle tissue samples was chronically up-regulated for the central pathways related to energy metabolism, including oxidative phosphorylation, lipid metabolism and supportive metabolic pathways. Up-regulation of these pathways was associated in particular with aerobic fitness and high HDL cholesterol levels. In fat tissue we found physical activity-associated increases in the expression of polyunsaturated fatty acid metabolism and branched-chain amino acid degradation gene sets both of which associated with decreased ‘high-risk’ ectopic body fat and plasma glucose levels. Consistent with other findings, plasma lipidomics analysis showed up-regulation of the triacylglycerols containing the polyunsaturated fatty acids. Our findings identified skeletal muscle and fat tissue pathways which are associated with the long-term physical activity and reduced cardio-metabolic disease risk, including increased aerobic fitness. In particular, improved skeletal muscle oxidative energy and lipid metabolism as well as changes in adipocyte function and redistribution of body fat are associated with reduced cardio-metabolic risk.

## Introduction

Increased or reduced risks for common chronic cardio-metabolic diseases are the result of complex molecular networks responding to genetic and environmental factors [1], [2]. A phenotype characterized by high physical activity and/or aerobic fitness predicts low cardio-metabolic morbidity and mortality more strongly than any other known biological risk factor [3]–[9]. These associations seem to be explained mechanistically via a complex network of pathways, including changes in body composition and serum cardio-metabolic risk factor levels [9], such as the high high-density lipoprotein cholesterol (HDL-C) among highly physically active individuals [10].

Skeletal muscles represent more than one-third of the body mass of a normal weight person and play an important role in the whole-body energy metabolism. When working vigorously, skeletal muscles strongly increase their oxidative activity from that of the resting level. The skeletal muscles also have a high capacity to adapt to changes in metabolic demand [11]. While many studies have been published on the effects of exercise training on athletic performance, the health-related effects of skeletal muscle metabolism has also received much attention, with findings indicating that molecular mechanistic networks in skeletal muscle may have an influence on cardio-metabolic risk factors [6], [12]–[14]. Fat tissue is an important energy store for endurance-type physical activities and its metabolism is linked to the aerobic metabolism of skeletal muscle and cardio-metabolic risk [13]. However, physical activity-induced changes in muscle and fat tissue-related complex molecular networks and their links with cardio-metabolic disease risk are not comprehensively understood [14].

As it is difficult to carry out very long-term randomized controlled exercise trials, and as observational population-based follow-ups may include genetic selection bias, we carried out within-pair analyses in middle-aged same-sex twin pairs identified on the basis of their long-term discordance for physical activity [15]–[17]. By studying twin pairs, we were able to control for childhood environment and partially for genetic liability. The aim of our co-twin control study with a 32-year-long follow-up was to investigate how gene expression profiles of skeletal muscle and fat tissue differ between physically inactive and active members of twin pairs and how these gene expression differences are associated with physical fitness and other cardio-metabolic risk factors. Our findings identify skeletal muscle as well as fat tissue pathways which are associated with the long-term physical activity and reduced cardio-metabolic disease risk, including the increase in aerobic fitness.

## Results

**Figure 1:**
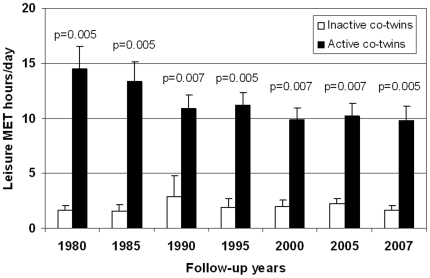
Follow-up physical activity discordance in the 10 twin pairs. Data is Mean ± SD. Calculation of mean MET discordance was based on a series of structured questions on leisure time physical activity and was quantified by calculation of the leisure activity metabolic equivalent [intensity x duration x frequency] expressed as a sum score of leisure time MET hours/day [3], [17].

Sixteen middle-aged (50–74 yrs) same-sex twin pairs discordant for physical activity for more than 30 years were identified from the *Finnish Twin Cohort* [15]. Ten twin pairs (Table 1, Figure 1, Table S1) volunteered to give muscle and fat biopsies for this study as in three pairs at least one twin had a chronic disease and in three pairs one or both co-twins refused.

**Table 1:** Characteristics of 10 twin pairs discordant for physical activity.

Characteristics	Inactive N = 10	Active N = 10	Mean Difference (95% CI)	p-Value
**Baseline (1975; self-reported)**				
Body height (cm) *(N = 9)*	172.7±9.4	170.3±9.2	2.3 (−2.6 to 7.3)	0.31
Body weight (kg)	67.8±18.6	63.7±10.0	4.1 (−7.6 to 15.8)	0.48
BMI (kg/m^2^) (*N = 9)*	22.6±3.7	22.2±1.8	0.4 (−2.7 to 3.5)	0.67
**Follow-up (2007; measured)**				
Body height (cm)	170.7±9.8	168.6±8.9	2.1 (−2.0 to 6.3)	0.28
Body weight (kg)	78.4±23.0	69.1±11.7	9.3 (−4.9 to 23.6)	0.14
BMI (kg/m^2^)	26.5±4.3	24.2±2.8	2.3 (−1.4 to 6.0)	0.20
Whole body fat percent (%) a	25.5±5.6	19.9±5.9	5.6 (1.2 to 10.1)	0.019
Visceral fat area (cm^2^) b	158.4±122.7	90.4±70.0	68.0 (−9.3 to 145.4)	0.037
IMAT area (cm^2^) c	11.4±5.7	7.5±4.2	3.9 (−0.7 to 8.6)	0.038
Estimated VO_2peak_ (ml/kg/min) d	28.3±3.6	33.0±5.0	−4.7 (−8.6 to −0.8)	0.023
Fasting plasma glucose (mmol/L)	5.3±1.3	4.7±0.6	0.6 (−0.3 to 1.4)	0.022
HOMA index	2.34±1.57	1.37±0.85	0.97 (−0.34 to 2.28)	0.059
Total cholesterol (mmol/L)	5.8±0.8	5.3±1.1	0.5 (−0.3 to 1.2)	0.24
HDL-C (mmol/L)	1.6±0.4	1.8±0.5	−0.2 (−0.3 to −0.01)	0.037
Triglycerides (mmol/L)	1.1±0.7	0.8±0.4	0.3 (−0.04 to 0.7)	0.059

BMI, Body mass index; IMAT, Intramuscular (extra myocellular) fat; HOMA index, (Fasting plasma glucose x Fasting plasma insulin)/22.5; HDL-C, High-density lipoprotein cholesterol.

a Measured by InBody (720) (Biospace, Korea) body composition analyzer [16].

b Measured by MRI [16].

c Cross-sectional intramuscular fat area of midthigh measured from MR-image [16].

d Calculated from symptom-limited maximal exercise test [15].

The active compared to inactive co-twins had higher peak oxygen uptake, a lower whole body fat percentage with lower ectopic ‘high-risk’ fat accumulation, higher HDL-C levels and lower fasting glucose and triglyceride levels (Table 1).

After normalizing the muscle gene expression data within pairs (normalization to the inactive twin) one-sample t-test discovered congruent lists of differentially expressed sequences: 45 sequences at P<0.001, 572 sequences at P<0.01 and 2829 sequences at P<0.05. Of the 45 sequences at P<0.001, 25 sequences were up-regulated and 20 sequences were down-regulated in the physically active co-twins (Table S2).

Pathway analysis using Gene Set Enrichment Analysis (GSEA) utilizing active vs. inactive co-twin gene expression ratios was performed on curated gene sets of canonical pathways containing 639 gene sets. For skeletal muscle the analysis yielded ten enriched gene sets with a FDR q-value <0.01. The most enriched gene sets in the active members of twin pairs were oxidative phosphorylation, valine, leucine and isoleucine degradation, ubiquinone biosynthesis and fatty acid metabolism (Table 2). Specific genes in the oxidative phosphorylation gene set encode NADH dehydrogenase (complex I), succinate dehydrogenase (complex II), cytochome c oxidase, H^+^ transport and ATP synthase (Figure S1). Genes in the valine, leucine and isoleucine degradation group were related to aldehyde dehydrogenase activity, branched-chain amino acid catabolic processes and steps of the mitochondrial fatty acid beta-oxidation pathway. The “leading-edge” genes (i.e. genes contributing to the enrichment scores of GSEA analysis) that were used to calculate the expression centroids are shown in Table S3. Many genes or gene sets encoding the steps of the mitochondrial electron transport chain were up-regulated in active compared to inactive co-twins (Table S2, Table S3).

**Table 2:** Gene sets up-regulated in skeletal muscle among active compared to inactive co-twins (GSEA analysis).

Gene Set Name	Up-regulated/Size	ES	NOM p-Value	FDR q-Value
HSA00190 Oxidative phosphorylation	51/111	0.272	<0.0001	<0.0001
HSA00280 Valine, leucine and isoleucine degradation	27/44	0.383	<0.0001	0.00036
Valine, leucine and isoleucine degradation	24/35	0.425	<0.0001	0.00047
HSA00130 Ubiquinone biosynthesis	7/8	0.786	<0.0001	0.0015
Propanoate metabolism	21/30	0.415	<0.0001	0.00015
HSA00071 Fatty acid metabolism	21/47	0.335	<0.0001	0.0021
HSA00650 Butanoate metabolism	20/45	0.329	<0.0001	0.0044
HSA00380 Tryptophan metabolism	32/60	0.277	0.0016	0.009
Fructose and mannose metabolism	11/24	0.422	<0.0001	0.0085
Glycolysis	17/52	0.285	0.0016	0.0091
HSA00641_3 Chloroacrylic acid degradation	9/15	0.496	<0.0001	0.013
HSA00220 Urea cycle and metabolism of amino groups	14/30	0.369	0.0017	0.013

ES, Enrichment score (the primary outcome of GSEA analysis); NOM p-value, Nominal p-value; FDR q-value, False discovery rate q-value

High correlation coefficients (*r*) and high coefficients of determinations (*R^2^*) were found between peak oxygen uptake and gene set centroids ranked by active-to-inactive ratio (Figure S2). Percentage of the total measured area of muscle cross-section graded to be most oxidative according to succinate dehydrogenase (SDH) staining (Figure S3) were also positively correlated with centroids ranked by the active-to-inactive ratio (Figure S2).

HDL-C was higher in the active compared to inactive co-twins (Table 1) and was significantly correlated with 9 out of 10 centroids of gene sets upregulated in active co-twins (Figure S4). Adjustment for gender changed the correlations only minimally. Each of the centroids of the first five gene sets explained the variation in HDL-C levels with coefficients of determination from 0.22 to 0.39. Also, intrapair differences of the gene set centroids correlated well with those of the HDL-C levels (Figure S4).

In order to investigate the metabolic and expression changes due to physical (in)activity in the adipose tissue as well, the global gene expression profiles were analyzed from the abdominal subcutaneous adipose tissue samples (taken on the same occasion as the muscle biopsies). Forty-seven sequences at P<0.001 (one-sample t-test after normalization of data), 401 sequences at P<0.01 and 2037 sequences at P<0.05 were differentially expressed between active and inactive. Of the 47 sequences at P<0.001, 16 sequences were up-regulated and 31 sequences were down-regulated in the physically active co-twins (Table S4). The most enriched gene sets in the active co-twins (Table 3) included valine, leucine and isoleucine degradation (related to aldehyde dehydrogenase activity, branched-chain amino acid catabolic processes and steps of the mitochondrial fatty acid beta-oxidation pathway, as found also in skeletal muscle), polyunsaturated fatty-acid (PUFA) metabolism and inflammatory processes. The “leading-edge” genes used to calculate expression centroids in fat tissue, are shown in Table S5. Interestingly, the gene set centroids most up-regulated in the active co-twins also had a very high correlation with reduced BMI, reduced visceral fat, reduced intramuscular but extracellular fat accumulation, reduced serum triglycerides, reduced plasma glucose and increased HOMA index (Table S6).

**Table 3:** Gene sets up-regulated (with FDR q-values ≤0.10) in subcutaneous abdominal fat tissue among active compared to inactive co-twins (GSEA analysis).

Gene set name	Up-regulated/Size	ES	NOM p-Value	FDR q-Value
IL2RB pathway	12/34	0.61	<0.0001	0.073
Valine, leucine and isoleucine degradation	26/35	0.58	<0.0001	0.10
HSA01040 Polyunsaturated fatty acid biosynthesis	10/14	0.67	<0.0001	0.077
HSA00280 Valine, leucine and isoleucine degradation	29/44	0.56	<0.0001	0.099
RECK pathway	4/9	0.72	0.001	0.089
Prostaglandin synthesis regulation	14/28	0.58	<0.0001	0.085
T cytotoxic pathway	2/11	0.68	0.002	0.087

ES, Enrichment score; NOM p-value, Nominal p-value; FDR q-value, False discovery rate q-value.

In order to link the findings from tissue-specific gene expression with systemic lipid metabolism, plasma lipidomics analysis was performed. Among all 16 twin pairs [15] nominally statistically significant differences between active compared to inactive co-twins were found in 14 of the 215 lipids identified (Table 4). Seven of the centroids of gene sets up-regulated in skeletal muscle samples in the active compared to inactive co-twins associated statistically significantly with ChoE (18:2) with coefficients of determination from 0.15 to 0.46 (Table S7).

**Table 4:** Lipids differing in plasma lipidomics between active and inactive co-twins^a^.

Lipid Name	FC	p-Value	FDR q-Value
ChoE (18:2)	1.47	0.0053	0.10
TG (58:10)	2.32	0.0056	0.10
TG (56:9)	2.03	0.0069	0.10
TG (50:5)	1.73	0.010	0.13
TG (58:7)	1.60	0.013	0.14
TG (58:9)	2.33	0.014	0.14
TG (49:3)	1.53	0.017	0.14
TG (47:0)	1.84	0.018	0.14
TG (56:8)	2.16	0.025	0.15
TG (58:8)	2.15	0.027	0.15
TG (53:5)	1.52	0.034	0.17
TG (54:6)	1.60	0.046	0.20
TG (56:7)	2.63	0.048	0.21
TG (54:7)	1.64	0.049	0.21

a n = 16 twin pairs.

FC, fold change (increased active/inactive ratio); FDR, False discovery rate.

## Discussion

**Figure 2:**
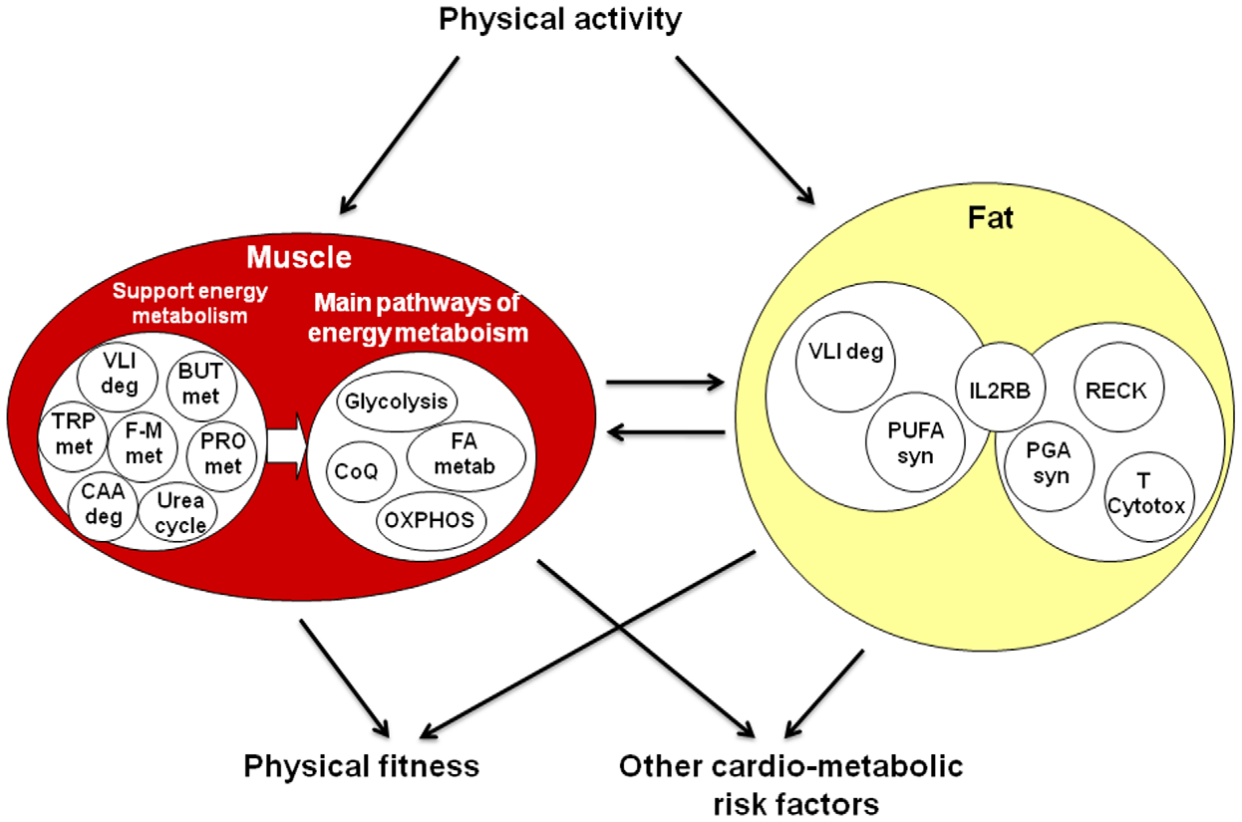
Up-regulated gene sets in muscle and fat tissue and their association with cardio-metabolic risk. Among the physically active members of twin pairs, as compared to their inactive co-twins, gene expression in the skeletal muscle was up-regulated for the central pathways of energy metabolism and supportive metabolic pathways related especially to the processes of oxidative energy production. In fat tissue the pathways were related e.g. to branched-chain amino acid degradation and PUFA synthesis. These metabolic changes were associated with decreased cardio-metabolic risk, including an increase in aerobic fitness. Centroids: OXPHOS, Oxidative phosphorylation; VLI deg, Valine, leucine and isoleucine degradation; CoQ, Ubiquinone biosynthesis; PRO met, Propanoate metabolism; FA metab, Fatty acid metabolism including mitochondrial β-oxidation and peroxisomal β-oxidation; BUT met, Butanoate metabolism; TRP met, Tryptophan metabolism; F-M met, Fructose and mannose metabolism; CAA deg, Chloroacrylic acid degradation; Urea cycle, Urea cycle and metabolism of amino groups; IL2RB, IL2RB pathway; PUFA syn, Polyunsaturated fatty acid biosynthesis; RECK, RECK pathway; PGA syn, Prostaglandin synthesis regulation; T Cytotox, T cytotoxic pathway.

Our study identifies the gene sets most up-regulated among highly and persistently physically active members of twin pairs compared to their inactive co-twins in skeletal muscle and fat tissue and gives further information about their association with cardio-metabolic risk factors (Figure 2).

Studying scattered individual genes and their function can only account for a small part of the phenomena underlying complex traits. Obesity, insulin resistance and advanced age as well as many chronic diseases are suggested to be related to reduced muscle mitochondrial function [18]. In our study the up-regulated gene sets in muscle and adipose tissue were partially the same and partially different although related to the same mechanisms. Together, our results show that long-term physical activity is associated with high oxidative capacity of skeletal muscle as seen by up-regulation of the genes encoding energy metabolism, oxidative phosphorylation and lipid metabolism. In particular, up-regulation of genes encoding the steps of the mitochondrial electron transport chain, which suggests enhanced oxidation capacity of the skeletal muscle (i.e. mitochondrial biogenesis) of the active co-twins, was seen [11], [19].

The complex mechanisms underlying the association between up-regulated skeletal muscle pathways and high HDL-C, which we observed at the systemic level, are to some extent unknown. It is worth noting that HDL-C has vasodilatatory effects [20] further enhanced by specific steroid-fatty acid components of HDL-C [21] which may contribute to the increased oxygen supply to muscles. Also, increase in the oxidative capacity of the mitochondrial apparatus switches fuel preference towards fatty acids which seems to accompany with increased cholesterol export.

Mechanistically, our study also supports earlier findings that increased branched-chain amino acid catabolism is linked to increased oxidative energy production, lower ectopic fat accumulation and lower insulin resistance [22]–[24]. Dysregulated branched-chain metabolism may make an independent contribution to development of insulin resistance and glucose intolerance, ultimately leading to type 2 diabetes [25]. In adipose tissue, expression of the oxidative pathway was previously found to be suppressed in the obese and poor fitness phenotype [26]. In monozygotic twin pairs discordant for obesity, differences in branched-chain amino acid catabolism and adipose tissue mitochondria count have also been observed [24].

Increased PUFA synthesis may contribute to increased fitness and reduced cardio-metabolic risk by contributing to membrane functions and by increasing peroxisomal beta-oxidation and further oxidative phosphorylation, both of which were seen to be higher among physically active co-twins compared to inactive on the basis of muscle gene expression.

In plasma lipidomics analysis the up-regulation of triacylglycerols containing the polyunsaturated fatty acids is consistent with increased PUFA synthesis in adipose tissue (Table 3) and elevated HDL-C (Table 1). These triglycerides were also positively associated with improved insulin sensitivity in an earlier study [27].

Interestingly, the gene set expression centroids most up-regulated in the adipose tissue of the active co-twins also had a very high correlation with reduced BMI, reduced visceral fat, reduced intramuscular but extracellular fat accumulation, reduced serum triglycerides, reduced plasma glucose and reduced HOMA index (Table S6). This is in line with the known effects of exercise training [9]. Physically inactive subjects were less insulin sensitive than active co-twins as the HOMA index tended to be higher in inactive co-twins. It is to note that muscle contraction stimulates translocation of Glut4 glucose transporter via an insulin-independent mechanism [28] but this insulin sensitizing mechanism of contractile activity is evident only during 24 hours after exercise [14]. Thus, our study investigated other non-acute mechanisms.

Our study also suggested physical-activity associated up-regulation of some other gene sets in adipose tissue (Table 4). Some of the genes up-regulated in the IL2RB pathway also seem to be linked to better membrane functions and insulin sensitivity. The RECK pathway, prostaglandin synthesis regulation and the T cytotoxic pathway may contribute among other things to regulation of inflammation, cell proliferation and vasoconstriction/dilatation (Table S5), but these findings need further confirmation.

### Strengths and limitations

In our study we employed a co-twin control design using twin pairs with 30-year discordance for physical activity habits. However, only moderate statistical power in our study means that there is a risk for type II error in particular in analyses of adipose tissue gene expression data. Comparing members of same-sex twin pairs takes into account effects of age and gender but it is to note that our material included both MZ and DZ pairs. Concerning individual-based correlations we have analyzed that the presented associations were not explained by gender. Dietary factors may also influence the findings of our study, but as there were only minor differences in nutrient intakes between active and inactive co-twins [29] it is very unlikely that dietary differences explain our findings. There were no significant differences in the smoking habits or use of alcohol between the active and inactive co-twins [15]. Also, as the trends between active and inactive co-twins usually were rather similar among DZ and MZ pairs, our findings seem not to be explained by sequence level genetic differences between co-twins. The carefully documented discordance in physical activity between co-twins was further confirmed by the differences in the co-twins tibial bone properties [30]. Related to the gene expression studies lack of protein-level analyses is a limitation. Although all subjects were free from cardio-metabolic diseases and other diseases affecting the ability to be physically active in 1975, very long-term physical activity may lead to different metabolic consequences, such as insulin resistance, which may have an effect on gene expression in muscle and adipose tissue. On the basis of our analyses on the associations between physical activity, gene expression and cardio-metabolic risk factors it seems that the observed up-regulations in skeletal muscle gene expression are not directly related to all of the cardio-metabolic risk factors but decreased fat accumulation and differences in body fat distribution may mediate the association between high physical activity and many of the other cardio-metabolic risk factors, including insulin resistance.

### Clinical conclusions

To make clinical conclusions a limitation of our study is the small sample size, which was because of our strict criterion for activity discordance to demonstrate reliably the effects of physical activity. Despite the fact that the studied persistent leisure-time physical activity levels (inactivity or activity) are more common in the population, it is less common that co-twins of a twin pair have persistently different activity levels. Unfortunately no data exists from large population samples to exactly describe how big proportion of individuals are persistently physically inactive or active during leisure time over a 30-year period according to our criteria. Thus, our study can be regarded as a model giving evidence on the associations between long-term physical activity vs. inactivity, gene expression and cardio-metabolic risk factors. Also, our study shows the associations between mitochondrial function, PUFA and branched-chain amino acid metabolism and occurrence of metabolic disorders. Interestingly, very similar associations were found when rats with high intrinsic aerobic capacity were compared to those with low capacity [31] suggesting that both inherited and acquired properties contribute to metabolic disease risk factors. So, they are good targets for future bio-marker research and possibly for drug development research. Our findings agree on the hypothesis that physical activity-associated increased skeletal muscle use and oxidative energy metabolism may contribute to decreased fat accumulation and changes in adipocyte function and redistribution of body fat, and further that these consequent changes in adipose tissue may have an effect on the development of insulin resistance.

## Methods

### Subjects

Sixteen middle-aged and older (50–74 yrs) same-sex twin pairs discordant for physical activity for more than 30 years were identified (TWINACTIVE study) from the *Finnish Twin Cohort*. For detailed subject recruitment and clinical assessments see Leskinen et al. [15]: http://www.atypon-link.com/AAP/doi/pdf/10.1375/twin.12.1.108. In brief, ten twin pairs of which 3 were monozygotic (2 female) and 7 dizygotic (2 female) pairs (Table 1) volunteered to give muscle and fat biopsies for this study as in three pairs at least one twin had a chronic disease and in three pairs one or both co-twins refused. Gene expression analyses on muscle tissue samples were successfully carried out for all of these twin pairs and on fat tissue samples for six complete pairs (2 monozygotic and 4 dizygotic pairs).

The identification process of participants was primarily carried out on the basis of physical activity data and the researchers were blinded to data on height, weight and other body composition characteristics [15], [17]. Discordance was based on a series of structured questions on leisure activity and physical activity during journeys to and from work. The leisure time (metabolic equivalent (MET)) index was calculated by assigning a multiple of the resting metabolic rate (intensity x duration x frequency) and expressed as a sum score of leisure time MET hours per day [3], [15], [17]. The discordance for physical activity was initially identified in the assessment carried out in 1975. The discordance for leisure-time activity was assessed in the following three stages. First, twin pairs discordant for physical activity in 1975 and also in the assessment carried out in 1981 were identified (165 out of 5663 twin pairs defined as healthy in 1981). Second, a retrospective follow-up interview on leisure activity (covering the years from 1980 to 2005 in 5-year intervals) was carried out. On the basis of these assessments 54 out of the 165 pairs were included for further studies [15]. Finally, 16 twin pairs fulfilled all the TWINACTIVE study inclusion criteria, volunteered to participate in the TWINACTIVE study measurements and were discordant for leisure-time physical activity on the basis of the detailed physical activity interview conducted in 2007 [15]. The ICC between the shorter MET index and the detailed 12-month physical activity MET index was 0.68 (P<0.001) for leisure time physical activity and 0.93 (P<0.001) for work journey.

Physical activity discordance during the follow-up period for the ten twin pairs included in this study is shown in Figure 1 and Table S1. Leisure time physical activity between the inactive and active members of the twin pairs differed in 1975, 1981 and at each of the 7 follow-up occasions, the mean difference between the co-twins amounting to 9.4 MET h/day (2.0±1.9 vs. 11.4±3.0, 95% CI 7.6 to 11.2, p = 0.005). However, the subjects were advised not to exercise vigorously (except for walking) during the morning and two days before both of their laboratory visit (one visit for clinical examinations including exercise tests and one visit for biopsy studies) as we investigated long-term adaptations to exercise [15].

### Other health habits

Smoking habits and use of alcohol, together with other confounders, were collected with diary, questionnaire and interview methods as described earlier [3], [15], [17]. Energy intake was assessed using a 5-day food diary [29].

### Muscle and adipose tissue needle biopsies

Tissue samples were taken after an overnight fast between 8 am and 10 am under local anaesthesia after skin cooling and disinfection. The muscle biopsy was taken from the mid-part of *m. vastus lateralis* defined as the midpoint between the greater trochanter and the lateral joint line of the knee using Bergström's needle (ø 5 mm) biopsy technique with suction, and a needle biopsy (12 G needle, ø 2 mm) of subcutaneous abdominal adipose tissue was taken at the level of the umbilicus. The samples were cleaned of any visible connective tissue and muscle samples were cleaned of any visible adipose tissue. One part of the biopsies was frozen in liquid nitrogen immediately after withdrawing from the needle and stored at -80°C until used for mRNA analysis. The second part of the muscle biopsy used for succinate dehydrogenase analysis was mounted transversely on a cork with Optimal Cutting Temperature compound (Tissue Tek™, Miles, Elkhart, In, USA; Sakura, Cat. # 4583), and frozen rapidly (10-15 sec) in 2-Methylbutane (isopentane) (Fluka, Cat. # 59080) precooled to −160°C in liquid nitrogen and stored at −80°C.

### Succinate dehydrogenase (SDH) staining

The activity of SDH in muscle cryosections was assessed histochemically [32]. The converted 8-bit images (range of gray-levels 0–255) from the stained sections were processed and analyzed using ImageJ software (NIH). An intensity threshold representing minimal intensity values corresponding to SDH activity was set manually and uniformly for all images (least oxidative 18–56; most oxidative 137–206). Finally, three intensity scaled fractions representing different level of oxidative capacities were expressed as the percentage of the total measured area.

### Gene-expression array

The RNA preparation, cRNA generation and microarray hybridization procedures were used as previously described [33]. In brief, Trizol-reagent (Invitrogen, Carlsbad, CA) was used to isolate total RNA from muscle biopsy samples of *m. vastus lateralis* homogenized on FastPrep FP120 apparatus (MP Biomedicals, Illkirch, France). From adipose tissue total RNA was isolated following needle suspension with Ambion's RNAqueous -Micro Kit (AM 1931, Applied Biosystems) according to manufacturer's instructions. Experion (Bio-Rad Laboratories, Hercules, CA) was used to inspect RNA concentration and quality. Only pure, good-quality RNA was used in the further analyses (260/280 ratio >1.8). An Illumina RNA amplification kit (Ambion, Austin, TX) was used according to the manufacturer's instructions to obtain biotinlabeled cRNA from 500 ng of total RNA. Experion was used to perform quality control after amplification. Hybridizations (one array per tissue) to Illumina HumanWG-6 v3.0 Expression BeadChips (Illumina Inc., San Diego, CA, USA) containing probes for 48803 transcripts, were performed by the Finnish DNA Microarray Center at Turku Center for Biotechnology according to the Illumina BeadStation 500x manual (Revision C). Six samples were hybridized on the same chip with twin and co-twin always on the same chip. Hybridized probes were detected with Cyanin-3-streptavidin (1 µg/ml, Amersham Biosciences, GE Healthcare, Uppsala, Sweden) using Illumina BeadArray Reader (Illumina Inc.) and BeadStudio v3 software (Illumina Inc.). Raw data ( =  average probe signals) were extracted using the numerical results with Illumina Bead Studio v3.0.19 software with default settings without any additional normalization. The background for each bead was estimated by calculating the average of the 5 dimmest pixels in the area around the bead in question, outliers of transcript replicates greater than 3 deviations from the replicate median were removed, unexpressed genes were not removed and no log-transformations were performed. Initial data analyses were performed with R software environment for statistical computing (http://www.R-project.org), including Bioconductor development software (http://www.bioconductor.org). The raw data of each chip were quantile-normalized with affy package of Bioconductor [34]. Data quality was assessed by calculating Pearson correlations and clustering. For pairwise analysis normalized data was exported to Excel and SPSS statistical package. Fold change (FC) between twin-pairs was calculated by dividing the normalized expression value (of each gene) of the active twin with the respective value of the inactive twin. Statistical analysis of this data was done using one-sample t-test (FC vs. 1). In both analyses, lists of genes at different significance levels (P<0.05, P<0.01 and P<0.001) were created. The gene expression data and the raw data sets have been deposited in the GEO database, accession number GSE20319 for skeletal muscle data and GSE20536 for adipose tissue data. MIAME guidelines were followed during array data generation, preprocessing, and analysis. The clustering of differentially expressed genes into functional groups and significance of their distribution among groups was estimated with Gene Set Enrichment Analysis (version 2.0; GSEA, http://www.broad.mit.edu/gsea/) [35]. A list of all transcripts on the chip ranked according to the inter-pair expression ratio was utilized in the GSEA analysis with 1000 “gene set” permutations. The “leading-edge” genes (i.e. genes contributing to enrichment scores of GSEA analysis) were used to calculate expression centroids. The mean centroid of each leading-edge subset was computed by normalizing the expression levels of all subset genes to a mean of zero (0) [36].

### Lipid risk factor and lipidomics analyses from serum and plasma

After overnight fast, a blood sample was drawn from an antecubital vein. Total cholesterol, triglycerides, and HDL-C were analyzed using VITROS DT60 (Chemistry System Ortho-Clinical Diagnostics, Inc., Rochester, NY, USA). Plasma glucose was determined using Biosen C-line (EKF-diagnostic, Magdeburg, Germany). Plasma lipidomics analysis was performed using ultra-performance liquid chromatography coupled to electrospray ionization mass spectrometry (UPLC-ESI-MS) as previously described in detail [37] with data processing using MZmine software version 0.60 [38].

### Ethical approval

This study was conducted according to good clinical and scientific practice/guidelines and the Declaration of Helsinki. All subjects provided written informed consent. The ethics committee of the Central Hospital of Central Finland approved our study plan on August 15, 2006.

### Other statistical analyses

Pairwise analyses were used to study differences between co-twins. The normality of variables was assessed by the Shapiro-Wilk test. Student's paired *t*-test was used for normally distributed variables and the Wilcoxon signed rank test for non-normally distributed variables. The symmetry tests (Stata version 8.0, www.stata.com) was used for the categorical variables. In the GSEA (1000 “gene set” permutations) and lipidomics analysis p-values were adjusted using False Discovery Rate (FDR) [35], [39]. Ninety-five percent confidence intervals (95% CI) were calculated for the absolute mean differences between the inactive and active co-twins. The Pearson correlation coefficient was used for the intrapair difference (absolute differences between pairs) correlations and for individual-based correlations between gene set centroids and cardio-vascular risk factors (supplementary files) when the number of observations of continuous variables was ≥10 and the examination of distributions were suggestive of normal distribution (all skewness values for cardio-vascular risk factors <1 and for gene set centroids <2.2). When calculating individual-based coefficient of determination, the within-pair dependency of twin individuals was taken into account using the cluster option of Stata [40]. The level of significance was set at p<0.05. Data were analyzed using SPSS 14.0, Stata 8.0 and R software [41].

## Supporting Information

Figure S1 Example of the enrichment plot of oxidative phosphorylation. Core enrichment genes on the right. Genes are presented in the order they were situated in the GSEA ranking list and affected to the enrichment score (the most up-regulated gene first, etc.).
(2.51 MB TIF)

Figure S2 Associations between the centroids of gene sets up-regulated in muscle in the active compared to inactive co-twins and maximal oxygen uptake levels (A) and the proportion of most oxidative muscle cross-section as determined by succinate dehydrogenase staining (B). r, Correlation coefficient; R2 and p-value from family cluster regression analysis. Centroids: OXPHOS, Oxidative phosphorylation; VLI deg, Valine, leucine and isoleucine degradation; CoQ, Ubiquinone biosynthesis; PRO met, Propanoate metabolism; FA metab, Fatty acid metabolism including mitochondrial β-oxidation and peroxisomal β-oxidation; BUT met, Butanoate metabolism; TRP met, Tryptophan metabolism; F-M met, Fructose and mannose metabolism; CAA deg, Chloroacrylic acid degradation; Urea cycle, Urea cycle and metabolism of amino groups.
(0.07 MB TIF)

Figure S3 Percentage distribution of the total measured area of muscle cross-section of different oxidative capacities according to succinate dehydrogenase staining. Data is Mean ± SD. The inactive vs. active differences in succinate dehydrogenase staining muscle cross-section percentages were statistically non-significant; least oxidative (p = 0.31), intermediate oxidative (p = 0.24) and most oxidative (p = 0.24) muscle cross-section percentage.
(0.07 MB TIF)

Figure S4 Associations between the centroids of gene sets up-regulated in the active compared to inactive co-twins and HDL-C levels. Individual-based (n = 20) correlation coefficients (r) and R2 and p-values from family cluster regression analysis are shown in panel A. Correlation plot between intrapair differences (IPD) in HDL-C and in centroid of oxidative phosphorylation (n =  10 pairs; panel B) and valine, leucine and isoleucine degradation (panel C). Centroids: OXPHOS, Oxidative phosphorylation; VLI deg, Valine, leucine and isoleucine degradation; CoQ, Ubiquinone biosynthesis; PRO met, Propanoate metabolism; FA metab, Fatty acid metabolism including mitochondrial β-oxidation and peroxisomal β-oxidation; BUT met, Butanoate metabolism; TRP met, Tryptophan metabolism; F-M met, Fructose and mannose metabolism; CAA deg, Chloroacrylic acid degradation; Urea cycle, Urea cycle and metabolism of amino groups.
(0.06 MB TIF)

Table S1 Physical activity MET-indices of the inactive and active members of the twin pairs during follow-up.
(0.07 MB DOC)

Table S2 Significantly regulated genes in muscle tissue with one-sample t-test p<0.001.
(0.08 MB DOC)

Table S3 Genes contributing to enrichment scores and calculation of expression centroids in muscle tissue.
(0.05 MB DOC)

Table S4 Significantly regulated genes in fat tissue with one-sample t-test p<0.001.
(0.08 MB DOC)

Table S5 Genes contributing to enrichment scores and calculation of expression centroids in fat tissue.
(0.05 MB DOC)

Table S6 Associations between the centroids of gene sets up-regulated in active vs. inactive co-twins in fat tissue and cardio-metabolic risk factors.
(0.05 MB DOC)

Table S7 Associations between the centroids of gene sets up-regulated in muscle tissue in the active compared to inactive co-twins and ChoE (18:2).
(0.05 MB DOC)
