# Supplementary figures and images for: Differences in Muscle and Adipose Tissue Gene Expression and Cardio-Metabolic Risk Factors in the Members of Physical Activity Discordant Twin Pairs
Source: PLoS One. 2010 Sep 16;5(9):e12609. doi: 10.1371/journal.pone.0012609 (PMC2940764; doi:10.1371/journal.pone.0012609)

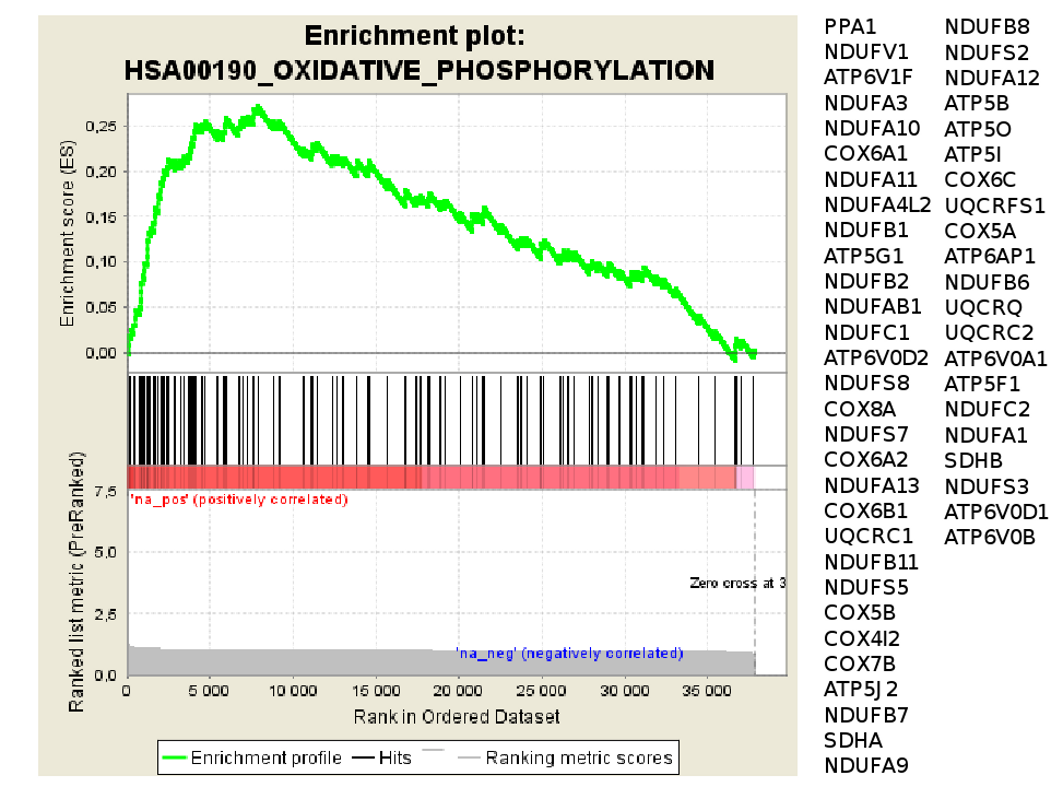

Supplement: Figure S1 — Example of the enrichment plot of oxidative phosphorylation. Core enrichment genes on the right. Genes are presented in the order they were situated in the GSEA ranking list and affected to the enrichment score (the most up-regulated gene first, etc.). (2.51 MB TIF) [file pone.0012609.s001.tif]

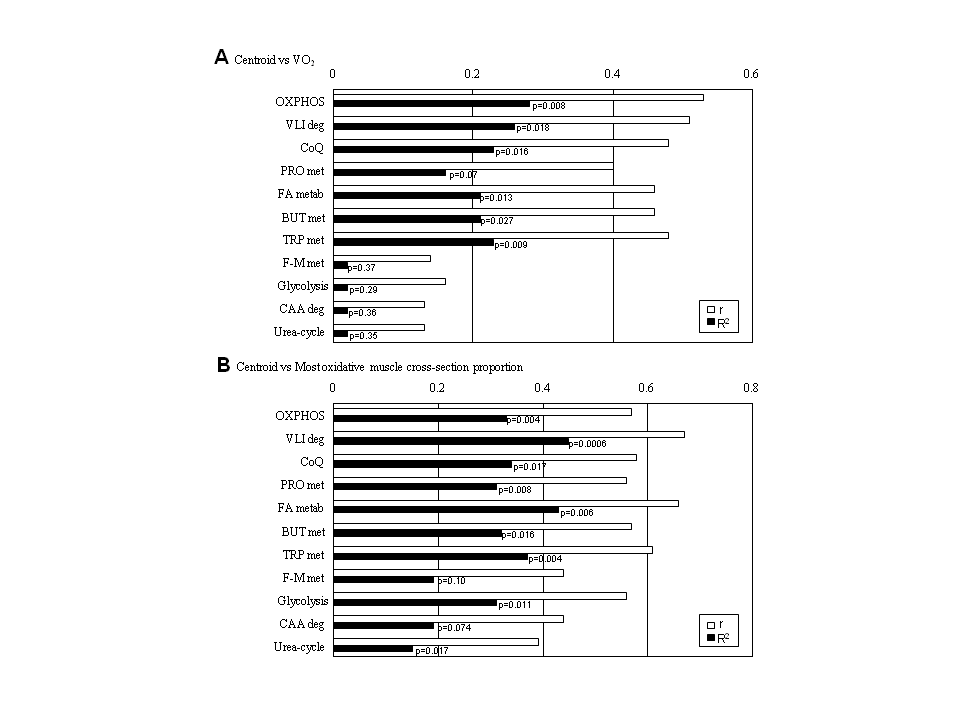

Supplement: Figure S2 — Associations between the centroids of gene sets up-regulated in muscle in the active compared to inactive co-twins and maximal oxygen uptake levels (A) and the proportion of most oxidative muscle cross-section as determined by succinate dehydrogenase staining (B). r, Correlation coefficient; R2 and p-value from family cluster regression analysis. Centroids: OXPHOS, Oxidative phosphorylation; VLI deg, Valine, leucine and isoleucine degradation; CoQ, Ubiquinone biosynthesis; PRO met, Propanoate metabolism; FA metab, Fatty acid metabolism including mitochondrial β-oxidation and peroxisomal β-oxidation; BUT met, Butanoate metabolism; TRP met, Tryptophan metabolism; F-M met, Fructose and mannose metabolism; CAA deg, Chloroacrylic acid degradation; Urea cycle, Urea cycle and metabolism of amino groups. (0.07 MB TIF) [file pone.0012609.s002.tif]

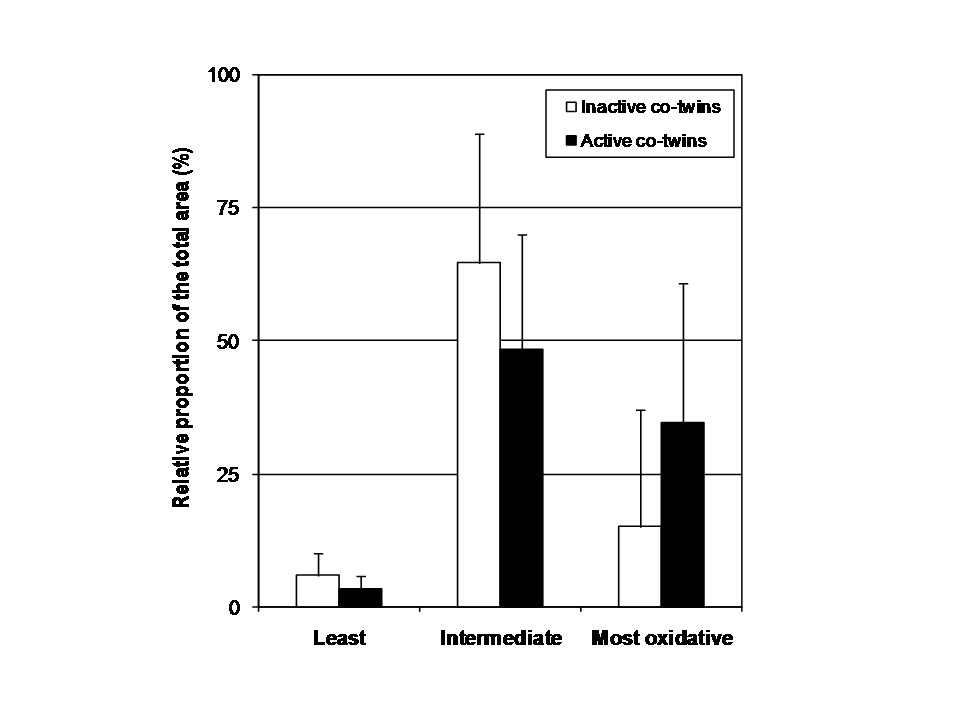

Supplement: Figure S3 — Percentage distribution of the total measured area of muscle cross-section of different oxidative capacities according to succinate dehydrogenase staining. Data is Mean ± SD. The inactive vs. active differences in succinate dehydrogenase staining muscle cross-section percentages were statistically non-significant; least oxidative (p = 0.31), intermediate oxidative (p = 0.24) and most oxidative (p = 0.24) muscle cross-section percentage. (0.07 MB TIF) [file pone.0012609.s003.tif]

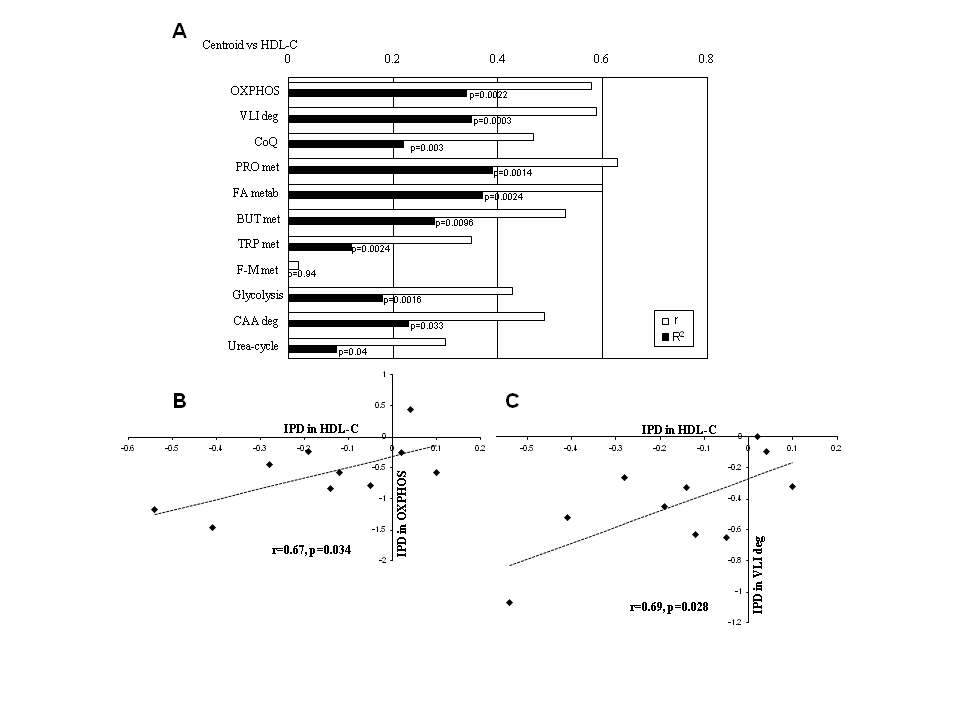

Supplement: Figure S4 — Associations between the centroids of gene sets up-regulated in the active compared to inactive co-twins and HDL-C levels. Individual-based (n = 20) correlation coefficients (r) and R2 and p-values from family cluster regression analysis are shown in panel A. Correlation plot between intrapair differences (IPD) in HDL-C and in centroid of oxidative phosphorylation (n = 10 pairs; panel B) and valine, leucine and isoleucine degradation (panel C). Centroids: OXPHOS, Oxidative phosphorylation; VLI deg, Valine, leucine and isoleucine degradation; CoQ, Ubiquinone biosynthesis; PRO met, Propanoate metabolism; FA metab, Fatty acid metabolism including mitochondrial β-oxidation and peroxisomal β-oxidation; BUT met, Butanoate metabolism; TRP met, Tryptophan metabolism; F-M met, Fructose and mannose metabolism; CAA deg, Chloroacrylic acid degradation; Urea cycle, Urea cycle and metabolism of amino groups. (0.06 MB TIF) [file pone.0012609.s004.tif]
