# Supplementary material for: Differences in Muscle and Adipose Tissue Gene Expression and Cardio-Metabolic Risk Factors in the Members of Physical Activity Discordant Twin Pairs
Source: PLoS One. 2010 Sep 16;5(9):e12609. doi: 10.1371/journal.pone.0012609 (PMC2940764; doi:10.1371/journal.pone.0012609)
Supplement: Table S1 — Physical activity MET-indices of the inactive and active members of the twin pairs during follow-up. (0.07 MB DOC) [file pone.0012609.s005.doc]

| Physical activity MET-indices of the inactive and active members of the twin pairs during follow-up. | | | | | | | | | |
| --- | --- | --- | --- | --- | --- | --- | --- | --- | --- |
| **Pair** | **Physical activity** | **1975** | **1980** | **1985** | **1990** | **1995** | **2000** | **2005** | **2007** |
| Pair 1 | Inactive | .03 | .60 | .60 | .60 | .23 | .40 | .40 | .61 |
|  | Active | 2.44 | 6.14 | 6.14 | 9.64 | 9.64 | 7.50 | 7.50 | 4.98 |
| Pair 2 | Inactive | .08 | 2.29 | 2.29 | .40 | .15 | .15 | 2.25 | 1.49 |
|  | Active | 3.75 | 12.83 | 12.50 | 7.50 | 7.50 | 7.50 | 12.50 | 11.73 |
| Pair 3 | Inactive | .01 | .10 | .10 | .10 | .10 | .80 | 1.60 | .83 |
|  | Active | 4.00 | 7.50 | 9.64 | 7.50 | 7.50 | 7.50 | 7.50 | 9.15 |
| Pair 4 | Inactive | .01 | .40 | .73 | .73 | 2.50 | 2.50 | 2.50 | 4.40 |
|  | Active | .60 | 16.58 | 16.58 | 16.58 | 17.30 | 8.55 | 13.55 | 4.48 |
| Pair 5 | Inactive | .11 | 2.59 | .80 | .80 | 1.60 | 1.60 | 1.93 | 2.20 |
|  | Active | 1.88 | 16.25 | 12.50 | 12.50 | 12.50 | 12.50 | 7.50 | 3.96 |
| Pair 6 | Inactive | .19 | 2.60 | 1.20 | 1.60 | 1.60 | 5.00 | 5.00 | 1.32 |
|  | Active | 1.30 | 27.08 | 27.08 | 6.58 | 8.55 | 4.80 | 4.80 | 7.90 |
| Pair 7 | Inactive | .08 | .80 | .80 | 1.50 | 1.50 | 1.50 | 1.50 | 3.64 |
|  | Active | 2.00 | 12.50 | 12.50 | 12.50 | 12.50 | 12.50 | 12.50 | 11.59 |
| Pair 8 | Inactive | .20 | 3.75 | 5.89 | 19.82 | 8.39 | 3.75 | 4.08 | 4.93 |
|  | Active | 3.13 | 16.25 | 16.25 | 16.25 | 16.25 | 16.25 | 16.25 | 15.69 |
| Pair 9 | Inactive | .20 | 2.94 | 2.94 | 2.94 | 2.94 | 3.74 | 2.65 | 1.92 |
|  | Active | 2.40 | 857 | 7.14 | 7.14 | 7.14 | 8.57 | 7.14 | 6.52 |
| Pair 10 | Inactive | .01 | .30 | .30 | .30 | .30 | .30 | .30 | .96 |
|  | Active | 2.40 | 21.17 | 12.83 | 12.83 | 12.83 | 12.83 | 12.83 | 7.54 |

MET-index: Calculation is based on a series of structured questions on leisure activity and physical activity during journeys to and from work. The leisure time (metabolic equivalent (MET)) index was calculated by assigning a multiple of the resting metabolic rate (intensity x duration x frequency) and expressed as a sum score of leisure time MET-hours per day. Calculation in 1975 is based on questionnaire reports, from 1980 through 2005 on retrospective interview and in 2007 on detailed interview of activities during past 12 months (for more details and references see Methods).
