# Supplementary material for: Differences in Muscle and Adipose Tissue Gene Expression and Cardio-Metabolic Risk Factors in the Members of Physical Activity Discordant Twin Pairs
Source: PLoS One. 2010 Sep 16;5(9):e12609. doi: 10.1371/journal.pone.0012609 (PMC2940764; doi:10.1371/journal.pone.0012609)
Supplement: Table S2 — Significantly regulated genes in muscle tissue with one-sample t-test p<0.001. (0.08 MB DOC) [file pone.0012609.s006.doc]

| Significantly regulated genes in muscle tissue with one-sample t-test p<0.001 | | | | |
| --- | --- | --- | --- | --- |
| **Accession** | **Symbol** | **p-Value** | **FC** | **Gene definition** |
| **Up-regulated in active twins** | | | | |
| NM_198893.1 | ZNF160 | 0.00004 | 1.14 | Homo sapiens zinc finger protein 160 (ZNF160), transcript variant 2, mRNA. |
| NM_001006115.2 | IHPK1 | 0.00013 | 1.08 | Homo sapiens inositol hexaphosphate kinase 1 (IHPK1), transcript variant 2, mRNA. |
| BM669906 |  | 0.00018 | 1.06 | UI-E-DW1-aha-a-03-0-UI.s1 UI-E-DW1 Homo sapiens cDNA clone UI-E-DW1-aha-a-03-0-UI 3, mRNA sequence |
| NM_016199.1 | LSM7 | 0.00019 | 1.13 | Homo sapiens LSM7 homolog, U6 small nuclear RNA associated (S. cerevisiae) (LSM7), mRNA. |
| NM_152383.4 | DIS3L2 | 0.00021 | 1.11 | Homo sapiens DIS3 mitotic control homolog (S. cerevisiae)-like 2 (DIS3L2), mRNA. |
| NM_032821.2 | HYDIN | 0.00024 | 1.07 | Homo sapiens hydrocephalus inducing homolog (mouse) (HYDIN), transcript variant 1, mRNA. |
| NM_173701.1 | WARS | 0.00028 | 1.15 | Homo sapiens tryptophanyl-tRNA synthetase (WARS), transcript variant 2, mRNA. |
| NM_007103.2 | NDUFV1 | 0.00036 | 1.21 | Homo sapiens NADH dehydrogenase (ubiquinone) flavoprotein 1, 51kDa (NDUFV1), mRNA. |
| DR033780 |  | 0.00040 | 1.06 | 5000EBA11 Fetal Brain 18 Homo sapiens cDNA 5, mRNA sequence |
| NM_016486.2 | TMEM69 | 0.00052 | 1.17 | Homo sapiens transmembrane protein 69 (TMEM69), mRNA. |
| AK125417 |  | 0.00056 | 1.08 | Homo sapiens cDNA FLJ43428 fis, clone OCBBF2027478 |
| NM_053050.2 | MRPL53 | 0.00060 | 1.18 | Homo sapiens mitochondrial ribosomal protein L53 (MRPL53), nuclear gene encoding mitochondrial protein, mRNA. |
| NM_012465.2 | TLL2 | 0.00061 | 1.08 | Homo sapiens tolloid-like 2 (TLL2), mRNA. |
| XM_927468.1 | LOC644295 | 0.00063 | 1.06 | PREDICTED: Homo sapiens similar to T25G3.1 (LOC644295), mRNA. |
| AI419775 |  | 0.00063 | 1.04 | tg39e08.x1 Soares_NFL_T_GBC_S1 Homo sapiens cDNA clone IMAGE:2111174 3, mRNA sequence |
| NM_024839.1 | RPP21 | 0.00078 | 1.10 | Homo sapiens ribonuclease P/MRP 21kDa subunit (RPP21), mRNA. |
| BQ013362 |  | 0.00079 | 1.09 | UI-1-BC1p-ayv-h-11-0-UI.s1 NCI_CGAP_Pl3 Homo sapiens cDNA clone UI-1-BC1p-ayv-h-11-0-UI 3, mRNA sequence |
| BU618600 |  | 0.00085 | 1.07 | UI-H-FH1-bfk-g-10-0-UI.s1 NCI_CGAP_FH1 Homo sapiens cDNA clone UI-H-FH1-bfk-g-10-0-UI 3, mRNA sequence |
| NM_080866.2 | SLC22A9 | 0.00086 | 1.07 | Homo sapiens solute carrier family 22 (organic anion transporter), member 9 (SLC22A9), mRNA. |
| XR_016431.1 | LOC642397 | 0.00088 | 1.05 | PREDICTED: Homo sapiens similar to WAS protein homology region 2 domain containing 1 (LOC642397), mRNA. |
| XM_928726.1 | LOC645718 | 0.00089 | 1.06 | PREDICTED: Homo sapiens hypothetical protein LOC645718 (LOC645718), mRNA. |
| XM_936519.1 | LOC647451 | 0.00092 | 1.07 | PREDICTED: Homo sapiens similar to heat shock protein 90Bf (LOC647451), mRNA. |
| BG231389 |  | 0.00096 | 1.08 | nai42a05.x1 NCI_CGAP_HN20 Homo sapiens cDNA clone IMAGE:4262673 3, mRNA sequence |
| BX116661 |  | 0.00097 | 1.07 | BX116661 NCI_CGAP_Kid8 Homo sapiens cDNA clone IMAGp998A094866, mRNA sequence |
| NM_145241.2 | WDR31 | 0.00098 | 1.10 | Homo sapiens WD repeat domain 31 (WDR31), transcript variant 3, mRNA. |
| **Down-regulated in active twins** | | | | |
| NM_001005861.1 | RYK | 0.00003 | 0.91 | Homo sapiens RYK receptor-like tyrosine kinase (RYK), transcript variant 1, mRNA. |
| NM_005493.2 | RANBP9 | 0.00010 | 0.87 | Homo sapiens RAN binding protein 9 (RANBP9), mRNA. |
| AA021278 |  | 0.00014 | 0.95 | ze66d06.s1 Soares retina N2b4HR Homo sapiens cDNA clone IMAGE:363947 3, mRNA sequence |
| NM_020875.1 | FRAS1 | 0.00020 | 0.95 | Homo sapiens Fraser syndrome 1 (FRAS1), transcript variant 3, mRNA. |
| AI125472 |  | 0.00021 | 0.91 | qd93f02.x1 Soares_testis_NHT Homo sapiens cDNA clone IMAGE:1737051 3, mRNA sequence |
| NM_022810.1 | SLC25A14 | 0.00026 | 0.94 | Homo sapiens solute carrier family 25 (mitochondrial carrier, brain), member 14 (SLC25A14), nuclear gene encoding mitochondrial protein, transcript variant short, mRNA. |
| NM_017798.2 | YTHDF1 | 0.00028 | 0.96 | Homo sapiens YTH domain family, member 1 (YTHDF1), mRNA. |
| AL040205 |  | 0.00031 | 0.92 | DKFZp434G0413_r1 434 (synonym: htes3) Homo sapiens cDNA clone DKFZp434G0413 5, mRNA sequence |
| NM_002958.3 | RYK | 0.00040 | 0.95 | Homo sapiens RYK receptor-like tyrosine kinase (RYK), transcript variant 2, mRNA. |
| AI890346 |  | 0.00042 | 0.92 | wm84b04.x1 NCI_CGAP_Ut2 Homo sapiens cDNA clone IMAGE:2442607 3, mRNA sequence |
| NM_014811.3 | KIAA0649 | 0.00045 | 0.88 | Homo sapiens KIAA0649 (KIAA0649), mRNA. |
| NM_001005185.1 | OR6N1 | 0.00051 | 0.92 | Homo sapiens olfactory receptor, family 6, subfamily N, member 1 (OR6N1), mRNA. |
| XM_941485.1 | LOC652147 | 0.00052 | 0.90 | PREDICTED: Homo sapiens similar to U5 snRNP-specific protein, 200 kDa (LOC652147), mRNA. |
| NM_002877.4 | RAD51L1 | 0.00053 | 0.91 | Homo sapiens RAD51-like 1 (S. cerevisiae) (RAD51L1), transcript variant 1, mRNA. |
| NM_002392.2 | MDM2 | 0.00058 | 0.91 | Homo sapiens Mdm2 p53 binding protein homolog (mouse) (MDM2), transcript variant MDM2, mRNA. |
| NM_177424.2 | STX12 | 0.00068 | 0.92 | Homo sapiens syntaxin 12 (STX12), mRNA. |
| NM_014779.2 | TSC22D2 | 0.00076 | 0.92 | Homo sapiens TSC22 domain family, member 2 (TSC22D2), mRNA. |
| NM_014344.2 | FJX1 | 0.00078 | 0.92 | Homo sapiens four jointed box 1 (Drosophila) (FJX1), mRNA. |
| XM_941499.1 | LOC652155 | 0.00086 | 0.92 | PREDICTED: Homo sapiens similar to Ig heavy chain V-I region HG3 precursor (LOC652155), mRNA. |
| NM_182811.1 | PLCG1 | 0.00099 | 0.92 | Homo sapiens phospholipase C, gamma 1 (PLCG1), transcript variant 2, mRNA. |

FC, Fold change
