# Supplementary material for: Differences in Muscle and Adipose Tissue Gene Expression and Cardio-Metabolic Risk Factors in the Members of Physical Activity Discordant Twin Pairs
Source: PLoS One. 2010 Sep 16;5(9):e12609. doi: 10.1371/journal.pone.0012609 (PMC2940764; doi:10.1371/journal.pone.0012609)
Supplement: Table S3 — Genes contributing to enrichment scores and calculation of expression centroids in muscle tissue. (0.05 MB DOC) [file pone.0012609.s007.doc]

| Genes contributing to enrichment scores and calculation of expression centroids in muscle tissue^a^ | |
| --- | --- |
| **Name** | **Genes** |
| Oxidative phosphorylation | PPA1, NDUFV1, ATP6V1F, NDUFA3, NDUFA10, COX6A1, NDUFA11, NDUFA4L2, NDUFB1, ATP5G1, NDUFB2, NDUFAB1, NDUFC1, ATP6V0D2, NDUFS8, COX8A, NDUFS7, COX6A2, NDUFA13, COX6B1, UQCRC1, NDUFB11, NDUFS5, COX5B, COX4I2, COX7B, ATP5J2, NDUFB7, SDHA, NDUFA9, NDUFB8, NDUFS2, NDUFA12, ATP5B, ATP5O, ATP5I, COX6C, UQCRFS1, COX5A, ATP6AP1, NDUFB6, UQCRQ, UQCRC2, ATP6V0A1, ATP5F1, NDUFC2, NDUFA1, SDHB, NDUFS3, ATP6V0D1, ATP6V0B |
| Valine, leucine and isoleucine degradation | BCAT2, IVD, ALDH6A1, OXCT1, ECHS1, ALDH9A1, AUH, EHHADH, HSD17B10, ALDH2, MCCC1, ALDH7A1, ALDH1B1, ACADS, HMGCL, HMGCS1, BCKDHA, HIBADH, ALDH3A2, ACAT2, ACAA1, PCCB, MCCC2, MCEE, PCCA, DLD, ACAT1, ACADSB, ACADL, ALDH1A2, SDS |
| Ubiquinone biosynthesis | COQ7, NDUFA13, NDUFB11, COQ2, COQ3, COQ5, NDUFA12 |
| Propanoate metabolism | LDHB, ALDH6A1, ECHS1, ALDH9A1, EHHADH, ALDH2, SUCLG1, ALDH1B1, ACADSB, ACADL, ALDH3A2, ACAT2, LDHC, PCCB, MLYCD, MCEE, PCCA, ACAT1, ALDH1A2, SDS, SUCLG2 |
| Fatty acid metabolism | ECHS1, ADH1A, ALDH9A1, EHHADH, HSD17B10, ALDH2, CPT2, ADHFE1, ALDH7A1, DCI, ACOX3, ALDH1B1, ACADS, GCDH, ACADSB, ADH6, ADH7, ACADL, CPT1B, ALDH3A2, ACAT2 |
| Butanoate metabolism | OXCT1, ECHS1, ALDH9A1, EHHADH, HSD17B10, ALDH2, AKR1B10, ALDH7A1, PDHA1, AACS, ALDH1B1, PPME1, ACADS, HMGCL, HMGCS1, ILVBL, AADAC, ACSM1, ALDH3A2, ACAT2 |
| Tryptophan metabolism | MAOA, ECHS1, ALDH9A1, EHHADH, HSD17B10, ALDH2, WARS, ALDH7A1, ALDH1B1, AOC3, CAT, GCDH, PRMT3, WBSCR22, KMO, WARS2, LCMT1, PRMT7, LNX1, ALDH3A2, ACAT2, ACMSD, AFMID, DDC, PRMT8, PRMT6, AANAT, ACAT1, INDOL1, OGDH,TPH1, INDO |
| Fructose and mannose metabolism | ALDOC, ALDOB, PFKFB3, FBP1, FBP2, PFKP, PMM1, PFKFB1, GMDS, HK2, PMM2 |
| Glycolysis | LDHB, ALDOC, ADH1A, ALDOB, ALDH9A1, ENO1, ALDH2, ADHFE1, FBP1, FBP2, PFKP, GPI, PDHA1, ALDH1B1, HK2, ADH6, ADH7 |
| Chloroacrylic acid degradation | ADH1A, ALDH9A1, ALDH2, ADHFE1, ALDH7A1, ALDH1B1, ADH6, ADH7, ALDH3A2 |
| Urea cycle and metabolism of amino groups | MAOA, ARG2, ALDH9A1, ALDH2, ACY1, ALDH7A1, SAT1, SMS, ALDH1B1, AOC3, GATM, OTC, CPS1, ALDH3A2 |

^a^Note the overlapping of genes, usually related to mitochondrial function. Genes are presented in the order they were in the GSEA ranking list and affected to the enrichment score (the most up-regulated gene first, etc.). Genes of valine, leucine and isoleucine degradation pathway represent a combination of two gene sets observed in GSEA analysis.
