# Supplementary material for: Differences in Muscle and Adipose Tissue Gene Expression and Cardio-Metabolic Risk Factors in the Members of Physical Activity Discordant Twin Pairs
Source: PLoS One. 2010 Sep 16;5(9):e12609. doi: 10.1371/journal.pone.0012609 (PMC2940764; doi:10.1371/journal.pone.0012609)
Supplement: Table S4 — Significantly regulated genes in fat tissue with one-sample t-test p<0.001. (0.08 MB DOC) [file pone.0012609.s008.doc]

| Significantly regulated genes in fat tissue with one-sample t-test p<0.001 | | | | |
| --- | --- | --- | --- | --- |
| **Accession** | **Symbol** | **p-Value** | **FC** | **Gene definition** |
| **Up-regulated in active twins** | |  |  |  |
| AA781984 |  | 0.000027 | 1.09 | ai78h04,s1 Soares_testis_NHT Homo sapiens cDNA clone 1376983 3, mRNA sequence |
| NM_001002916,2 | H2BFWT | 0.000072 | 1.09 | Homo sapiens H2B histone family, member W, testis-specific (H2BFWT), mRNA, |
| NM_001033016,1 | LOC613206 | 0.00012 | 1.13 | Homo sapiens myeloproliferative disease associated tumor antigen 5 (LOC613206), mRNA, |
| NM_018963,3 | BRWD1 | 0.00015 | 1.05 | Homo sapiens bromodomain and WD repeat domain containing 1 (BRWD1), transcript variant 1, mRNA, |
| NM_000436,3 | OXCT1 | 0.00026 | 1.11 | Homo sapiens 3-oxoacid CoA transferase 1 (OXCT1), nuclear gene encoding mitochondrial protein, mRNA, |
| XM_927557,1 | LOC644412 | 0.00035 | 1.10 | PREDICTED: Homo sapiens similar to Sodium/hydrogen exchanger 3 (Na(+)/H(+) exchanger 3) (NHE-3) (Solute carrier family 9 member 3) (LOC644412), mRNA, |
| NM_199361,1 | TPD52L2 | 0.00039 | 1.09 | Homo sapiens tumor protein D52-like 2 (TPD52L2), transcript variant 2, mRNA, |
| BG217842 |  | 0.00046 | 1.13 | RST37556 Athersys RAGE Library Homo sapiens cDNA, mRNA sequence |
| BQ007653 |  | 0.00051 | 1.08 | UI-H-EI0-ayh-k-15-0-UI.s1 NCI_CGAP_EI0 Homo sapiens cDNA clone IMAGE:5838854 3, mRNA sequence |
| NM_018946.2 | NANS | 0.00070 | 1.08 | Homo sapiens N-acetylneuraminic acid synthase (sialic acid synthase) (NANS), mRNA. |
| NM_175609.1 | ARFGAP1 | 0.00075 | 1.14 | Homo sapiens ADP-ribosylation factor GTPase activating protein 1 (ARFGAP1), transcript variant 2, mRNA. |
| NM_001037234.1 | TMEM75 | 0.00075 | 1.13 | Homo sapiens transmembrane protein 75 (TMEM75), mRNA. |
| NM_024759.1 | NPAL2 | 0.00083 | 1.09 | Homo sapiens NIPA-like domain containing 2 (NPAL2), mRNA. |
| AW298110 |  | 0.00084 | 1.15 | UI-H-BW0-ajs-c-11-0-UI.s1 NCI_CGAP_Sub6 Homo sapiens cDNA clone IMAGE:2732924 3, mRNA sequence |
| BG184196 |  | 0.00088 | 1.13 | RST3117 Athersys RAGE Library Homo sapiens cDNA, mRNA sequence |
| NM_012390.3 | SMR3A | 0.00090 | 1.05 | Homo sapiens submaxillary gland androgen regulated protein 3A (SMR3A), mRNA. |
| **Down-regulated in active twins** | |  |  |  |
| NM_032260.2 | RGPD5 | 0.000026 | 0.90 | Homo sapiens RANBP2-like and GRIP domain containing 5 (RGPD5), transcript variant 2, mRNA. |
| NM_207289.1 | AAA1 | 0.000066 | 0.94 | Homo sapiens AAA1 protein (AAA1), transcript variant VII, mRNA. |
| NM_016074.2 | BOLA1 | 0.00014 | 0.89 | Homo sapiens bolA homolog 1 (E. coli) (BOLA1), mRNA. |
| AA086324 |  | 0.00014 | 0.84 | zn61c08.s1 Stratagene muscle 937209 Homo sapiens cDNA clone IMAGE:562670 3, mRNA sequence |
| NM_031947.2 | SLC25A2 | 0.00017 | 0.89 | Homo sapiens solute carrier family 25 (mitochondrial carrier; ornithine transporter) member 2 (SLC25A2), nuclear gene encoding mitochondrial protein, mRNA. |
| AK091889 |  | 0.00018 | 0.93 | Homo sapiens cDNA FLJ34570 fis, clone KIDNE2008072 |
| DB341503 |  | 0.00022 | 0.94 | DB341503 TESTI4 Homo sapiens cDNA clone TESTI4041860 3, mRNA sequence |
| NM_199246.1 | CCNG1 | 0.00023 | 0.92 | Homo sapiens cyclin G1 (CCNG1), transcript variant 2, mRNA. |
| NM_000129.3 | F13A1 | 0.00027 | 0.63 | Homo sapiens coagulation factor XIII, A1 polypeptide (F13A1), mRNA. |
| AI634678 |  | 0.00029 | 0.88 | wa07h04.x1 NCI_CGAP_Kid11 Homo sapiens cDNA clone IMAGE:2297431 3, mRNA sequence |
| NM_003147.4 | SSX2 | 0.00037 | 0.89 | Homo sapiens synovial sarcoma, X breakpoint 2 (SSX2), transcript variant 1, mRNA. |
| NM_013997.1 | TAC1 | 0.00037 | 0.93 | Homo sapiens tachykinin, precursor 1 (substance K, substance P, neurokinin 1, neurokinin 2, neuromedin L, neurokinin alpha, neuropeptide K, neuropeptide gamma) (TAC1), transcript variant gamma, mRNA. |
| NM_002013.2 | FKBP3 | 0.00045 | 0.91 | Homo sapiens FK506 binding protein 3, 25kDa (FKBP3), mRNA. |
| NM_145300.1 | LOC200420 | 0.00047 | 0.84 | Homo sapiens LOC200420 (LOC200420), mRNA. |
| NM_018116.2 | MSTO1 | 0.00047 | 0.67 | Homo sapiens misato homolog 1 (Drosophila) (MSTO1), mRNA. |
| BX093286 |  | 0.00050 | 0.86 | BX093286 NCI_CGAP_Lu5 Homo sapiens cDNA clone IMAGp998P244067 ; IMAGE:1604231, mRNA sequence |
| AL044394 |  | 0.00056 | 0.91 | DKFZp434D022_s1 434 (synonym: htes3) Homo sapiens cDNA clone DKFZp434D022 3, mRNA sequence |
| NM_003060.2 | SLC22A5 | 0.00060 | 0.76 | Homo sapiens solute carrier family 22 (organic cation transporter), member 5 (SLC22A5), mRNA. |
| NM_003135.1 | SRP19 | 0.00062 | 0.86 | Homo sapiens signal recognition particle 19kDa (SRP19), mRNA. |
| CB047286 |  | 0.00064 | 0.92 | NISC_gg01h01.x1 NCI_CGAP_Kid11 Homo sapiens cDNA clone IMAGE:3253464 3, mRNA sequence |
| NM_004763.3 | ITGB1BP1 | 0.00072 | 0.79 | Homo sapiens integrin beta 1 binding protein 1 (ITGB1BP1), transcript variant 1, mRNA. |
| NM_145312.2 | ZNF485 | 0.00072 | 0.87 | Homo sapiens zinc finger protein 485 (ZNF485), mRNA. |
| XM_940214.1 | LOC137107 | 0.00078 | 0.91 | PREDICTED: Homo sapiens similar to ribosomal protein L10a (LOC137107), mRNA. |
| NM_016346.2 | NR2E3 | 0.00081 | 0.95 | Homo sapiens nuclear receptor subfamily 2, group E, member 3 (NR2E3), transcript variant 1, mRNA. |
| X93500 |  | 0.00084 | 0.91 | H.sapiens mRNA for upstream hIL-4 primer for unknown sequence (166bp) |
| NM_002264.1 | KPNA1 | 0.00088 | 0.94 | Homo sapiens karyopherin alpha 1 (importin alpha 5) (KPNA1), mRNA. |
| NM_020056.2 | HLA-DQA2 | 0.00089 | 0.90 | Homo sapiens major histocompatibility complex, class II, DQ alpha 2 (HLA-DQA2), mRNA. |
| NM_144504.1 | F11R | 0.00093 | 0.82 | Homo sapiens F11 receptor (F11R), transcript variant 5, mRNA. |
| NM_001001933.1 | LHX8 | 0.00094 | 0.92 | Homo sapiens LIM homeobox 8 (LHX8), mRNA. |
| NM_020409.2 | MRPL47 | 0.00095 | 0.80 | Homo sapiens mitochondrial ribosomal protein L47 (MRPL47), nuclear gene encoding mitochondrial protein, transcript variant 1, mRNA. |
| XM_931830.1 | LOC643813 | 0.00095 | 0.93 | PREDICTED: Homo sapiens hypothetical protein LOC643813 (LOC643813), mRNA. |

FC, Fold change
