# Supplementary material for: Differences in Muscle and Adipose Tissue Gene Expression and Cardio-Metabolic Risk Factors in the Members of Physical Activity Discordant Twin Pairs
Source: PLoS One. 2010 Sep 16;5(9):e12609. doi: 10.1371/journal.pone.0012609 (PMC2940764; doi:10.1371/journal.pone.0012609)
Supplement: Table S5 — Genes contributing to enrichment scores and calculation of expression centroids in fat tissue. (0.05 MB DOC) [file pone.0012609.s009.doc]

| Genes contributing to enrichment scores and calculation of expression centroids in fat tissue^a^ | |
| --- | --- |
| **Name** | **Genes** |
| IL2RB pathway | JAK1, SOS1, IRS1, MYC, FOS, CFLAR, STAT5A, SYK, STAT5B, PPIA, BCL2L1, CRKL |
| Valine, leucine and isoleucine degradation | SDS, ALDH1A2, ALDH2, EHHADH, IVD, ACADS, BCAT1, OXCT1, ACADSB, ALDH6A1, ACADM, ACADL, BCKDHA, PCCA, HIBADH, AOX1, MUT, BCKDHB, ACAT1, MCCC2, ACAT2, ECHS1, PCCB, MCCC1, HADHB, ALDH9A1, HADH, AUH, ALDH7A1, HSD17B10, BCAT2, HSD17B4, ACAA1, DLD |
| Polyunsaturated fatty acid biosynthesis | FADS1, FASN, SCD, PECR, ELOVL2, FADS2, ELOVL5, HSD17B12, GPSN2, ACAA1 |
| RECK pathway | MMP9, TIMP3, TIMP4, MMP14 |
| Prostaglandin synthesis regulation | EDN1, ANXA3, HPGD, ANXA4, TBXAS1, ANXA5, PTGER2, SCGB1A1, ANXA8, S100A6, ANXA6, PTGS1, PTGER1, PTGIR |
| T cytotoxic pathway | ITGB2, THY1 |

^a^Genes are presented in the order they were situated in the GSEA ranking list and affected to the enrichment score (the most up-regulated gene first, etc.). Genes of valine, leucine and isoleucine degradation pathway represent a combination of two gene sets observed in GSEA analysis.
