# Supplementary material for: Differences in Muscle and Adipose Tissue Gene Expression and Cardio-Metabolic Risk Factors in the Members of Physical Activity Discordant Twin Pairs
Source: PLoS One. 2010 Sep 16;5(9):e12609. doi: 10.1371/journal.pone.0012609 (PMC2940764; doi:10.1371/journal.pone.0012609)
Supplement: Table S6 — Associations between the centroids of gene sets up-regulated in active vs. inactive co-twins in fat tissue and cardio-metabolic risk factors. (0.05 MB DOC) [file pone.0012609.s010.doc]

| Associations between the centroids of gene sets up-regulated in active *vs*. inactive co-twins in fat tissue and cardio-metabolic risk factors | | | | | |
| --- | --- | --- | --- | --- | --- |
| **Gene set centroid** | **Visceral fat area** | **IMAT**  **area** | **fS-Trigly** | **fP-Gluk** | **HOMA index** |
|  | **r** | | | | |
|  | **R^2^** | | | | |
|  | **p** | | | | |
| IL2RB pathway | -0.86 | -0.72 | -0.68 | -0.91 | -0.87 |
|  | 0.74 | 0.52 | 0.46 | 0.82 | 0.76 |
|  | <0.001 | 0.013 | 0.015 | <0.001 | <0.001 |
| Valine, leucine and isoleucine degradation | -0.82 | -0.65 | -0.76 | -0.82 | -0.74 |
|  | 0.68 | 0.43 | 0.57 | 0.67 | 0.54 |
|  | 0.001 | 0.029 | 0.005 | 0.001 | 0.006 |
| Polyunsaturated fatty acid biosynthesis | -0.76 | -0.63 | -0.60 | -0.75 | -0.77 |
|  | 0.58 | 0.40 | 0.36 | 0.57 | 0.59 |
|  | 0.004 | 0.037 | 0.041 | 0.005 | 0.003 |

BMI, Body mass index; IMAT, Intramuscular (extracellular) fat area; HOMA index, (Fasting plasma glucose x Fasting plasma insulin)/22.5

Individual-based associations (n=12 individuals): r, Correlation coefficient between individuals; R^2^ and p-value from family cluster regression analysis
