# Supplementary material for: Differences in Muscle and Adipose Tissue Gene Expression and Cardio-Metabolic Risk Factors in the Members of Physical Activity Discordant Twin Pairs
Source: PLoS One. 2010 Sep 16;5(9):e12609. doi: 10.1371/journal.pone.0012609 (PMC2940764; doi:10.1371/journal.pone.0012609)
Supplement: Table S7 — Associations between the centroids of gene sets up-regulated in muscle tissue in the active compared to inactive co-twins and ChoE (18:2). (0.05 MB DOC) [file pone.0012609.s011.doc]

| Associations between the centroids of gene sets up-regulated in muscle tissue in the active compared to inactive co-twins and ChoE (18:2) | | | |
| --- | --- | --- | --- |
| **Gene set centroid** | **r** | **R^2^** | **p-Value** |
| Oxidative phosphorylation | 0.33 | 0.11 | 0.17 |
| Valine, leucine and isoleucine degradation | 0.38 | 0.14 | 0.066 |
| Ubiquinone biosynthesis | 0.12 | 0.01 | 0.52 |
| Propanoate metabolism | 0.46 | 0.21 | 0.023 |
| Fatty acid metabolism | 0.62 | 0.39 | 0.0016 |
| Butanoate metabolism | 0.62 | 0.38 | 0.010 |
| Tryptophan metabolism | 0.54 | 0.29 | 0.011 |
| Fructose and mannose metabolism | 0.39 | 0.15 | 0.023 |
| Glycolysis | 0.54 | 0.29 | 0.013 |
| Chloroacrylic acid degradation | 0.68 | 0.46 | 0.0050 |
| Urea cycle and metabolism of amino groups | 0.41 | 0.17 | 0.041 |

r, Pearson correlation coefficient; R^2^ and p-value from family cluster regression analysis
